# Social determinants of health equity for people with pain: from understanding to action

**DOI:** 10.1097/PR9.0000000000001364

**Published:** 2025-12-23

**Authors:** Emily Walker, Emma L. Karran, Matthew D. Jones, Mitchell T. Gibbs, Saurab Sharma

**Affiliations:** a School of Health Sciences, Faculty of Medicine and Health, UNSW Sydney, Sydney, Australia; b Centre for Pain IMPACT, Neuroscience Research Australia, Sydney, Australia; c IIMPACT in Health, University of South Australia, Kaurna Country, Adelaide, South Australia, Australia; d Pain Management and Research Centre, Royal North Shore Hospital, Northern Sydney Local Health District, Sydney, Australia; e Pain Management Research Institute, Kolling Institute, Faculty of Medicine and Health, The University of Sydney and Northern Sydney Local Health District, Sydney, Australia

**Keywords:** Chronic pain, Social determinants of health, Health equity, Pain management, Clinical care

## Abstract

**Objective:**

This article explores the role of social determinants of health in pain care, highlighting their contribution to health inequities and offering strategies for clinicians to respond to these factors in clinical practice.

**Methods:**

This clinical update aligns with the 2025 IASP Global Year, which focuses on pain management and education in low- and middle-income countries, as well as low-income settings and priority populations in high-income countries.

**Results:**

Social determinants of health—defined as the social, economic, and environmental conditions shaping people's health outcomes and care experiences—follow a social gradient, and disproportionately affect those facing disadvantage and multiple forms of marginalisation. They play a central role in creating health inequities, which are systematic, avoidable, and unfair differences in health between groups. Social determinants of health are upstream influences that create downstream impacts, including individual social risk factors and health-related social needs. Addressing these influences in pain care requires a multi-level approach that considers broader structural conditions alongside individual contexts. Clinicians can take meaningful action by screening for social factors, tailoring care to individuals' social needs, connecting patients with resources, establishing links within and outside the healthcare system, advocating both at the individual and system level, and critically reflecting on their biases, social identity, and the structural forces that shape health.

**Conclusion:**

Social determinants of health are central drivers of disparities in health outcomes and pain care. Although clinicians alone cannot address structural injustice, they play a crucial role in recognising and responding to social conditions that influence care, especially for those most affected by inequities.

## Abstract 2

Equitable pain care requires integrating awareness of social conditions and structural inequities with clinician reflection and coordinated action across healthcare, communities, and policy.

**Key Points**

- People's opportunities to live healthy lives are shaped by a wide range of social, economic, and environmental conditions—known as the social determinants of health.
- Pain outcomes, experiences, and management are shaped by these social conditions, yet challenges remain in identifying how this awareness can enhance routine clinical care.
- Unjust and unequal exposure to adverse social determinants of health contributes to avoidable health inequities, particularly among marginalised and socioeconomically disadvantaged groups.
- These inequities are produced and maintained by systems and structures that unequally distribute power, resources, and opportunities.
- Clinicians can play a critical role in recognising and responding to social factors that influence how individuals experience and manage pain.
- Achieving equity in pain care requires coordinated upstream and downstream action across healthcare, community, and policy sectors—not only to support individual needs, but to challenge and dismantle the structural conditions that sustain inequity.

## 1. Introduction

Chronic pain is a major global health challenge with individual, societal, and health system consequences. It is the leading cause of disability worldwide and contributes the most to the global burden of disease.^61^ Chronic pain conditions vary widely in type and occur across the life course.^31,146^ Despite decades of research and evolving treatment approaches, many people continue to live with chronic pain that disrupts daily activities, work, and quality of life.^75,111,127,165,177^

Chronic pain disproportionately affects people from low socioeconomic conditions. Its prevalence and burden is higher in people from low- and middle-income countries (LMICs) compared with individuals in affluent countries.^87,130,180^ Chronic pain not only affects adults but also has a similar prevalence in children and adolescents, with 1 in 5 people affected.^31^ In LMICs, prevalence is projected to continue increasing due to population growth, changing disease patterns, conflict, and climate change.^40,84,106,153^ Although research is beginning to document the clinical and economic impacts of pain globally, significant gaps remain because of underrepresentation of LMIC populations, language and publication bias, and methodological inconsistencies.^157^

Within-country differences also exist. For example, in high-income countries (HICs), socioeconomically disadvantaged groups (eg, Indigenous peoples, culturally diverse communities, refugees, people from rural and remote settings) are disproportionately affected by chronic pain.^84,97^ For example, Indigenous populations experience greater prevalence of pain than non-Indigenous people, and disparities exist between rural and remote areas compared to urban areas.^89,103,105^ Although individual countries are primarily responsible for their population's health, cross-border factors such as trade, migration, pharmaceutical access, and climate change significantly influence pain burden and care. This highlights the importance of shared global responsibility, global health governance, and international cooperation.^116,139^

Although the biopsychosocial nature of pain is widely accepted, challenges remain in applying it comprehensively in research and practice.^92,124^ In particular, the social dimensions of pain remain underconsidered in research, education, assessment, and management.^92,124,159^ When considered, social factors tend to focus on close relationships like those with friends or family, whereas broader social contexts shaping people's lives receive less attention. This may reflect a Western tendency to separate health from social environments.^169^ Yet for those living with pain, the social, emotional, and medical domains are deeply intertwined. Failure to account for this interconnectedness limits understanding of individuals' lived experiences and constrains the development of equitable models of care.^124,169^ This article builds on the International Association for the Study of Pain (IASP)'s Global Year focus on pain in low/middle-income settings,^1^ aiming to highlight the role of social determinants in achieving equitable pain care, identify research and clinical challenges to achieving this, and offer practical solutions to pain clinicians.

### 1.1. Social determinants of health

Social determinants of health (SDoH) are the societal conditions that shape individuals' social position and influence their access to power, money, resources, and health outcomes.^67^ These conditions include the environments in which people are born, grow, live, work, and age, as well as broader structural factors like economic systems, institutional policies, and societal norms.^15,67^ Cultural and systemic influences such as racism, sexism, ageism, and ableism play a critical role in shaping these conditions and affecting health.^67^

Health outcomes tend to follow a social gradient, where the lower an individual's income, access to healthcare, or educational attainment, the poorer their health.^67,125,126^ These conditions are therefore critical in creating health inequities—systematic, avoidable, and unfair differences in health between groups.^119^ Although groups are often defined by individual characteristics, such as ethnicity, gender, or income, it is not these characteristics themselves that determine health outcomes, but how social, structural, and systemic conditions interact with them.^67^ For example, being Indigenous or from a nondominant culture is not itself a SDoH. Rather, the interaction between historical and ongoing racism, discrimination, and limited availability and access to culturally safe healthcare predispose these communities to poorer health outcomes.^84^ In pain care, acknowledging social factors and their influence on health inequity shifts the focus from a purely biomedical or individual lens to one that understands pain as a socially situated experience shaped by broader systems and structures.^67,92,124,137^

The lens of upstream and downstream factors helps explain how SDoH impact individuals' opportunities to lead healthy lives. Upstream factors are political and structural determinants, such as policies that govern access to education, affordable housing, secure employment, and healthcare. This then flows downstream to shape individual behaviours, opportunities, and potentially biological outcomes, such as increased inflammation among people in lower socioeconomic positions.^10,113,174^ These upstream conditions give rise to social risk factors, which are specific adverse social conditions such as housing instability or social isolation, that increase the likelihood of poor health.^4^ Social risk factors may manifest as health-related social needs, practical barriers that affect individuals' ability to manage their health, including lack of access to safe housing, healthy food, or reliable transportation.^29^ Overall, SDoH are upstream structural determinants requiring societal or policy level interventions, whereas social risk factors and health-related social needs are the downstream, individual-level factors that clinicians can identify and respond to.^4^

Although SDoH, social risk factors, and health-related social needs are often used interchangeably, these are distinct concepts and distinguishing them is essential.^115^ Recognising this upstream–downstream continuum clarifies clinicians' role in addressing the social dimensions of chronic pain. At the population level, health equity can be achieved by addressing SDoH and at the individual level, it can be achieved by addressing social needs.^107,132^ Using this lens in pain care can enable clinicians to consider contextually informed and equitable treatment approaches, potentially contributing to more holistic care that is responsive to individual needs and circumstances.

### 1.2. How do the social determinants of health affect health and pain?

Social determinants of health shape the prevalence, experience, and outcomes of health.^16,67,108,116^ They are suggested to influence health and pain outcomes more than either genetic factors or healthcare access.^8,36,57,84,115^ Of the factors that can be changed to improve how long and well people live, about 40% are social and economic factors.^83^ Social factors interact bidirectionally with pain across the life course, influencing pain onset, prevalence, prognosis, severity, management, and outcomes.^84,92^ For example, socioeconomic position, educational attainment, and employment conditions are consistently linked to pain prevalence, physical function, and outcomes.^50,97,127^ Income and education have been shown to predict chronic pain and physical function.^154^ People with lower socioeconomic position and less education are more likely to experience adverse low back pain outcomes than those with higher socioeconomic position.^97^ They are also more likely to experience chronic pain, greater pain severity, and higher levels of pain-related disability.^50,102,165^ Other social factors such as occupation, geographical location, social exclusion, social support, trauma, violence, stigma, and gender also affect chronic pain prevalence, intensity, disability, management, and outcomes including quality of life.^5,16,50,53,69,78,92,101,112,145,150,170,176,180^

Intersectionality is an important consideration in understanding SDoH. It describes how marginalisation occurs through the interaction of multiple social identities (eg, ethnicity/race, class, gender) and systems of oppression.^47,48^ This lens highlights how systems of oppression like racism, colonialism, capitalism, and ableism shape exposure to SDoH, unequal allocation of resources, influence pain care, and perpetuate disparities in pain outcomes.^77,92,113,122,170^ For example, racial and ethnic minorities often experience greater pain burdens yet are less likely to receive adequate or appropriate pain assessment and treatment.^73,84,122^ Among Indigenous populations, musculoskeletal pain and headaches are more prevalent and often co-occur with cardiovascular and mental health conditions.^89^ These disparities reflect structural inequities such as economic disadvantage, limited access to culturally safe care, and social exclusion, which are shaped by the intersecting and ongoing impacts of colonisation, systemic discrimination, and suppression of self-determination.^32,54,65,84^ These interrelated factors demonstrate how intersectionality explains not only who is affected by pain disparities but also how overlapping systems of oppression shape their experiences of health, illness, and care.

Beyond outcomes, SDoH also shape people's experiences within pain care. Qualitative research highlights how poverty, racism, trauma, and housing instability affect treatment engagement, access, therapeutic relationship, and the feasibility of treatment plans.^14,170,171^ A common theme is the misalignment between clinician recommendations and patients circumstances, leading to patient disappointment, mistrust, frustration, and disengagement.^14,84,114,170,171^ People from marginalised communities often describe feeling stigmatised, dismissed or labelled as “difficult” or “noncompliant”, particularly when social conditions limit their ability to follow treatment advice.^134,168,170^ Together, this evidence shows how social factors not only shape health, pain prevalence, and outcomes, but also pain experiences and management.

## 2. Discussion

### 2.1. Research challenges, gaps, updates, and recommendations

Attention to the role of SDoH in the pain field is limited, impeding progress towards a greater understanding of pain-related health inequities and how to respond to them.^124,168^ A lack of robust primary data from LMICs forces reliance on modelling rather than local evidence to estimate pain burden.^23,66^ These risks generate incomplete or inaccurate projections that fail to reflect local contexts, potentially influencing policy decisions and resulting in costly, ineffective, or harmful interventions.^23,66^ Clinical trials also struggle to meaningfully involve end users, including people with lived pain experience and clinicians, with tokenism persisting.^30,104,109,136,161,164^ Barriers to genuine involvement include limited resources, lack of awareness of the value of participatory approaches, and ongoing epistemic injustice that undermines the knowledge and expertise of people with lived experience. When social factors are assessed and addressed, they are often inconsistently defined, reduced to interpersonal issues like social support, or conflated with the individual subjective experience.^92,124,135,170^ Moreover, the term “psychosocial” is frequently reduced to psychological constructs (eg, catastrophising),^91^ overlooking broader social influences.^38,124^

Clinical trials can further reinforce and accentuate inequities in pain care. Interventions commonly target behavioural modification to improve individual-level outcomes (eg, pain severity), often overlooking the structural and contextual barriers that shape peoples' ability to make healthy choices.^37,109,128^ Social determinants of health are rarely incorporated into research frameworks, and trials often exclude participants with comorbid mental health conditions, language or literacy barriers, factors that disproportionately affect marginalised populations.^6,93,104^ Clinical trials on exercise, an effective and recommended treatment for chronic pain, rarely report on social risk factors, limiting understanding around their influence on trial participation and outcomes.^11^ Moreover, clinical trials rarely collect and report data on sociodemographic characteristics related to SDoH or evaluate implementation factors such as reach and context.^93,113,157^ This limits identification of relevant treatment effect modifiers and relevance of findings, risking the development of inappropriate or unfeasible interventions for diverse populations.^93,113,157^ Finally, clinical research on social screening is limited by small sample sizes, lack of comparison groups, high heterogeneity in study designs, and insufficient validation especially across diverse populations, reducing the reliability and generalisability of findings.^59,94^ These issues perpetuate cycles of inequity in research, raising questions about how findings can meaningfully be applied across varied clinical and social contexts.

Despite challenges, pain research is increasingly recognising the importance of social factors. Researchers are now guided to report participants' sociodemographic characteristics more comprehensively across factors relevant to health equity.^96^ A recent global initiative used a consensus process to determine what equity-relevant data is most important to collect in pain research,^95^ with potential benefits including understanding study generalisability, facilitating subgrouping according to socially stratifiable characteristics, and equity considerations at all stages of the research process. Recommendations for reporting equity, diversity, and inclusivity in pain research are available,^140^ and equity extensions to reporting guidelines aim to improve health research practices more broadly.^55,172,173^ In addition, national standards and programs are prioritising meaningful patient and public involvement.^39,72,163^ These efforts support a more inclusive and context-sensitive evidence base that can better inform equitable pain care.

Future research could assess how social needs screening affects outcomes, test equity-oriented interventions and those targeting structural inequities, and integrate inclusion of social factors into clinical guidelines.^67,92,152^ Greater attention to how research questions are framed, what resources are available, and how to serve groups in proportion to need may improve the relevance of clinical research from an equity perspective.^81,118^ A first step could involve training researchers in equity-oriented frameworks (e.g., decolonial, critical theory, and disability justice) and embedding evaluation strategies to identify barriers, facilitators, and strategies to ensure interventions are equitable, sustainable, contextually relevant, and effective for diverse populations.^21,81,88,95,129^ Normalising research methods where the collection of sociodemographic data are comprehensive and harmonised, and where the study design, measurement, evaluation, and reporting are carried out through an equity lens, is essential.^92,95,96^ This includes appropriate collection and reporting of sample characteristics and disaggregation of data where necessary. Co-designing pain research and interventions with communities is critical to ensure they reflect local needs and values of diverse populations.^13,35,52,92,120^ Particular considerations include centring the voices of those living with pain, especially people at the intersection of multiple forms of oppression.^92,104^

### 2.2. Addressing social factors in practice: challenges and strategies

Addressing the SDoH in pain care requires coordinated action across multiple levels, from clinicians and communities to researchers and policymakers.^107,110,152^ Although clinicians cannot address systemic issues like poverty and racism within their day-to-day practice, they hold a critical frontline position that enables them to identify and respond to the social factors that shape an individual's pain experience. However, challenges remain in determining how this awareness can enhance routine clinical care and how clinicians can specifically address social factors.

Pain care practices often diverge from guideline-based recommendations globally.^155,179^ A biomedical paradigm remains dominant across healthcare systems and in clinical practice,^23,68,123,156^ contributing to overdiagnosis and overtreatment, particularly in LMICs.^2,3,155^ Although clinicians recognise the impact of social factors, many report feeling underprepared, unsupported, or constrained in addressing them because of structural barriers such as restrictive funding models, limited resources and training, and patient financial limitations.^42,134,135,160,166,171^

Clinicians often lack confidence in identifying social factors.^79^ They report uncertainty around scope of practice, particularly regarding how and when to address social factors.^160^ Diverging perspectives exist, where some clinicians view social factors as outside their clinical scope, whereas others worry about ethical breaches if they cannot help with identified issues.^25^ Social context is therefore infrequently raised or discussed in practice.^58^ Excluding social factors from clinical reasoning risks misinterpreting patient behaviour, such as attributing “nonadherence” to a lack of motivation or discipline on the individual's part.^92^ This reflects “healthism”—the belief that health is solely the result of individual choices.^46^ This view disregards how social factors constrain and shape peoples' options for pain prevention, treatment, and management, and may risk clinicians delivering care that patients find ineffective, inappropriate, and not equity-oriented.^12,44,92^

Equity-oriented care is a potential way to integrate social needs into clinical practice. It seeks to create safe and respectful environments by intentionally addressing the social, political, and economic factors that shape health, access to services, and outcomes.^20,63^ It involves tailoring care to individual needs, priorities, histories, and contexts, and has the potential to optimise patient outcomes such as quality of life, and confidence in managing and preventing health problems.^63^ Although questions around professional scope remain,^160^ professional standards across disciplines (eg, physiotherapy, psychology, general practice, and exercise physiology) commonly promote the inclusion of social and cultural factors in communication, assessment, and management.^26,60,76,147,158^ Equity-oriented interventions that incorporate trauma- and violence-informed, contextually-tailored, and cultural safety components have been shown to shift clinicians' awareness, understanding, confidence, skills, and practices.^20^ Therefore, integrating social factors into clinical care is not only within scope but can be critical for delivering equitable care.

So, what does equity-oriented care look like in practice? Many frameworks, tools, and networks are being developed to support the integration of social care into clinical settings.^29,34,131,148^ The National Academies of Medicine proposes 5 key actions (5As): Awareness, Adjustment, Assistance, Alignment, and Advocacy^131^ (Box 1). Clinical implementation will vary by setting, population, and available resources^131^ and may require considerations of patients' cultural and social contexts.^143,151^

Box 1. **Five key actions for social care integration**

• Awareness: Identify individuals' social risks (eg, limited transportation to pain clinic).

• Adjustment: Tailor treatment plans in response to barriers (eg, offer telehealth when transport is an issue).

• Assistance: Link individuals to resources (eg, transport vouchers).

• Alignment: Establish links with community services and other healthcare professionals to coordinate care (eg, coordinate with social workers to ensure transport options are known and accessible for all individuals).

• Advocacy: Use clinical insights to highlight when structures and systems create barriers to equitable care. Push for systems that make equitable care possible (eg, work to promote policies that change the transportation infrastructure within the community).

Awareness starts with recognising social factors as integral to health. Although social factors may not always be modifiable, their potential to influence pain, its treatment, and outcomes can at least be considered and discussed.^63,138^ Screening for social factors can be conducted by clinician or through self-report, such as through screening tools like PRAPARE^175^ or open-ended questions during history taking.^9,29,34,58,82^ Asking about factors that may affect pain management can elicit meaningful information that improves care relevance and guides management decisions.^44^ For example, clinicians may ask about access to safe walkable parks for exercise, financial constraints affecting ability to afford allied health services, or difficulty getting transport to the pain clinic. A single question like “Do you (ever) have difficulty making ends meet at the end of the month?” is a good predictor of poverty^18^ and can serve as a starting point to discuss how social and economic factors may influence patient's pain and their ability to engage in treatment. To avoid stereotyping, all patients could be offered screening, with confidentiality, safety, sensitivity, and consent prioritised.^9,64^ Screening alone, however, is insufficient without consequent action, such as referral pathways or support systems like community support groups or social prescribing.^9,144^ Clinicians who know how to ask about social needs are more likely to help their patients manage them^134^; and patients are more confident in the screening process if they trust their healthcare providers, understand the reason for screening, and know that the results will lead to practical help or support.^9^ Toolkits like CLEAR support clinicians in identifying, discussing, and responding to social needs, including guidance on language and referral.^34,134^ There are also resources that provide guidance on how to select an appropriate screening.^82^

Adjustment involves tailoring care to peoples' social context. Integrating considerations of socioeconomic conditions into clinicians' treatment planning can be an important practice for addressing health inequity.^9,24,58^ Challenges such as limited access to healthy food, a lack of opportunity to exercise, or difficulty paying for medications may limit the ability to deliver guideline-based pain management, if not considered or addressed before making treatment recommendations.^25^ Furthermore, tailoring may involve integrating patient's cultural and religious practices into pain management strategies or involving community/family support systems to reduce isolation and the impacts of stigma.^58^

Assistance and Alignment involve establishing connections within and outside the healthcare system, including multidisciplinary teams, community-based resources, organisations, and agencies.^7,9,25^ To bridge clinical and social care HICs are adopting models like social prescribing and “link worker” roles to use the strengths and resources available at interpersonal, community, and societal levels.^33,41,70,86,92,142,162^ In pain care, the “link worker” model embeds a staff member within clinical teams who facilitates care plans and connects individuals to community resources and support services.^162^ Examples include local walking or running programs and programs that teach cooking healthy low-cost meals,^25,142^ which can support pain self-management strategies such as activity pacing, engagement in exercise, and lifestyle changes. In some settings, multidisciplinary teams are sharing social power, with physicians, counsellors, nurses, social workers, community members, and others, engaging in shared decision-making to address both pain management and the social factors influencing it.^20,90^

Community-based pain management programs provide another practical avenue to address SDoH by offering accessible care for individuals from diverse backgrounds.^167^ Typically delivered at low or no cost with multidisciplinary support, these programs aim to empower people to understand their pain, equip them with self-management tools, and improve social connections and quality of life through education and individualised support.^167^ Community-based initiatives can also provide holistic peer support for culturally meaningful pain care, particularly for populations experiencing health inequities.^90^ In LMICs, where pain services are often fragmented or unavailable, community-led, context-adapted initiatives may be most important.^19,152^ Examples include training nonspecialist or lay health workers to deliver contextually appropriate care based on local resources and pain management needs.^99,152^

Advocacy can involve speaking on behalf of individuals to ensure they receive appropriate accommodations, or at broader levels, where clinicians contribute to public discourse or policy development.^25,51^ The CLEAR toolkit outlines ways clinicians can engage in advocacy, such as getting involved with community leaders, raising awareness on how social conditions harm patients, and partnering with local support resources and advocacy groups.^34^ Clinicians, as health experts, are well placed to speak to and engage with community representatives and medical organisations about the needs of their patients.^25^ For example, they can highlight how insecure work or housing instability may exacerbate chronic pain and limit engagement with care. Clinicians can also support political action by voting and engaging in collective movements that prioritise the social conditions shaping health.^7,51,133^ Importantly, as clinicians are often time poor, identifying ways in which they can sustainably advocate is necessary.^25^

Other practical steps within the clinic involve using inclusive language and displaying visible signs of inclusivity for LGBTQI + communities,^17^ displaying welcome signs in multiple languages,^63^ offering options to gender match clinician and patient,^178^ understanding culture-specific nonverbal cues,^178^ and recognising Indigenous and other non-Western forms of knowledge and culturally grounded practices.^52,65,84,178^ These practices aim to create culturally safe and inclusive environments, which may improve patient trust, therapeutic relationship, and engagement in pain management.

Equity-oriented pain care also requires clinicians to develop critical consciousness, an awareness of one's social context and reflection on the societal-level drivers of oppression and privilege.^77^ This involves engaging in critical self-reflection, where clinicians examine how their social identities, assumptions, and biases shape their ability to provide equitable pain care.^49,120,141^ Clinicians can inadvertently exacerbate patient challenges through implicit biases, questioning the legitimacy of individuals' pain, and perpetuating stigmatisation.^14,58,62,160,170^ Research suggests that biases and assumptions influence clinical interactions, patient trust, engagement, and access to pain care.^44,62,73,74^ Striving to understand the complexities of culture, the histories of marginalisation, the safety needs of patients, and the ways discrimination, stereotyping, and stigma affect pain experiences and healthcare engagement may be necessary for equitable pain care.^44,45,63,170,178^ Critical self-reflection is ongoing, requiring recognition of how power, privilege, and marginalisation operate within health systems and clinical interactions^58,120^ and their potential impact on pain experience, management and outcomes.^14,92,113,170^

Clinicians have identified multiple barriers to addressing social factors in practice, including lack of knowledge, skills, and time, inadequate payment models, a lack of information about referrable programs and services available, and feeling underprepared and unsupported.^25,43,98,100,160,171^ A lack of services and supports in the community in many settings (particularly in rural and remote communities), a lack of integration between healthcare sector and social services, and a lack of research on effective interventions serve to compound the difficulties of identifying appropriate care pathways.^25,98,100^ Importantly, challenges to clinician well-being in practice, such as compassion fatigue, feelings of powerlessness when faced with patients' social challenges, vicarious trauma, and burnout must be recognised.^20,100^

Several facilitators may help overcome these challenges. These include adopting interdisciplinary team models of care, supportive funding structures, clinically relevant information on local services, strong links with community, supervision, education, and research that demonstrates efficacy within the clinical environment.^25,98^ On a service level, clinicians have expressed that flexible business models, such as offering group sessions, telehealth, or tiered pricing, have been useful in tailoring care to peoples' socioeconomic circumstances.^166^ Organisations can also assess the inclusiveness of their services through tools like the Equity-Oriented Health Care Scale.^22^ Clinician well-being may be enhanced by connecting with like-minded peers, who can provide support and a place to share best practice information.^20,25^ These recommendations highlight the importance of individual, structural and relational support to embed equity-oriented approaches into pain care.

Clinicians may benefit from trainings, readings, and part-taking in organisations. A list of resources is provided in the Optional Further Reading List. On a broader level, university curricula and professional development must also evolve to include practical, context- and culture-sensitive strategies to help clinicians navigate social factors safely and effectively.^85,143,144,151,152^ Education is particularly important in LMICs where structural barriers are prominent.^27,71,84^ Clinicians can look for education that involves discussions linking educational content with the clinician's and patient's sociopolitical and community context.^20,21^ Furthermore, seeking facilitated opportunities to engage in communities experiencing disadvantage may offer meaningful collaborations and learning opportunities.^25,28^ Having opportunities for the process of experiential learning, reflection, and adjustment of practice may also be beneficial.^20,121^

### 2.3. Policy implications

Reducing health inequity requires policy that addresses the “causes of the causes”.^117^ Meeting individuals' social needs is necessary and can be life-saving, but addressing SDoH requires structural changes designed to create opportunities for health for all, in proportion to need.^118^ Policy can create and maintain conditions which result in health inequity, and it also holds the potential to prevent these disparities.^149^ As such, health is political and addressing upstream determinants, such as employment and education, are critical to reducing pain burden.^67,80^ Specific policy recommendations have been detailed elsewhere.^19,56,67,80,125,152^ These recommendations include, but are not limited to, adopting a “Health in All Policies” framework that draws attention to how decisions made outside of the health sector affect health,^67,116^ prioritising a health systems strengthening approach, partnering with global and local organisations, implementing universal health coverage, and prioritising pain in policy within LMICs.^19,152^

## 3. Conclusion

This clinical update explores how SDoH, social risk factors, and health-related needs shape people's outcomes, experiences, and management in pain care. The SDoH disproportionately impact marginalised groups through intersecting systems of oppression, resulting in health inequities. Although clinicians cannot directly address structural inequities in practice, they can engage meaningfully with individuals' social contexts to provide equity-oriented care. To do so, we recommend (1) screening for social factors; (2) tailoring care to individuals' social context; (3) linking individuals to resources; (4) establishing links within and outside the healthcare system; (5) advocating at individual and system levels; and (6) reflecting on how one's social identity, power, assumptions, and biases shape equitable care. Education, peer support, and building relationships with local communities may be key to implementing these recommendations. Addressing SDoH, social risk factors, and health-related needs requires action across individual, institutional, and systemic levels to support more equitable pain care for all, especially for those most affected.

## Disclosures

The authors have no conflict of interest to declare.

## Further Reading List

Blanket Exercise—An experiential workshop exploring the history of colonisation and its ongoing effects: kairoscanada.org/blanket-exercise.

San’yas Indigenous Cultural Safety Training—Online training to improve care for Indigenous people: sanyas.ca.

NCCIH Cultural Safety Training and Resources—Tools and publications supporting culturally safe Indigenous health care: nccah-ccnsa.ca.

Equity-Oriented Health Care Framework—Provides strategies for enacting equity-oriented healthcare in clinical practice: Browne AJ, Varcoe C, Ford-Gilboe M, Wathen CN. EQUIP healthcare: a framework for enhancing equity-oriented primary health care. *CMAJ* 2016;188:E474–83.

Clinical Tools to help with social determinants in practice—Andermann A. Taking action on the social determinants of health in clinical practice: a framework for health professionals. *CMAJ* 2016;188:E474–E483.

The Coin Model of Privilege and Critical Allyship—A framework to understand how unearned advantage operates and how to act in solidarity: Nixon SA. The coin model of privilege and critical allyship: implications for health. *BMC Public Health* 2019;19:1637.

FOR-EQUITY General Resources—A hub of videos, readings, and learning tools on intersectionality, racism, and social justice: forequity.uk/general-resources.

Health Inequalities Assessment Toolkit (HIAT)—A practical guide for integrating health equity considerations into research, service planning, and evaluation: forequity.uk/hiat.

EQUIP Health Care—Research, frameworks, and tools for implementing equity-oriented care in clinical practice: equiphealthcare.ca/resources.

Toolkits for Equity (C4DISC)—Resources on inclusive practice and equity in scholarly and professional settings: c4disc.org/toolkits-for-equity.

American Board of Family Medicine—Equity Certification Activities: theabfm.org/certification-activities.

Justice focused organisation—https://synergyforjustice.org/.

## References

[1] 2025 IASP Global Year-Pain Management, Research and Education in Low-and Middle-Income Settings. International Association for the Study of Pain (IASP), 2025. Available at: https://www.iasp-pain.org/advocacy/global-year/pain-management-research-and-education-in-low-and-middle-income-settings/. Accessed June 4, 2025.

[2] Albarqouni L Arab-Zozani M Abukmail E Greenwood H Pathirana T Clark J Kopitowski K Johansson M Born K Lang E Moynihan R. Overdiagnosis and overuse of diagnostic and screening tests in low-income and middle-income countries: a scoping review. BMJ Glob Health 2022;7:e008696. 10.1136/bmjgh-2022-008696 PMC9442491 36316027

[3] Albarqouni L Palagama S Chai J Sivananthajothy P Pathirana T Bakhit M Arab-Zozani M Ranakusuma R Cardona M Scott A Clark J Smith CF Effa E Ochodo E Moynihan R; the Overdiagnosis and Overuse of Healthcare Services in LMICs Network. Overuse of medications in low- and middle-income countries: a scoping review. Bull World Health Organ 2023;101:36–61D. 10.2471/BLT.22.288293 PMC9795388 36593777

[4] Alderwick H Gottlieb LM. Meanings and misunderstandings: a social determinants of health lexicon for health care systems. Milbank Q 2019;97:407–19. 10.1111/1468-0009.12390 PMC6554506 31069864

[5] Allen SF Gilbody S Atkin K van der Feltz-Cornelis C. The associations between loneliness, social exclusion and pain in the general population: a N = 502,528 cross-sectional UK biobank study. J Psychiatr Res 2020;130:68–74. 10.1016/j.jpsychires.2020.06.028 32791383

[6] Amundsen PA Evans DW Rajendran D Bright P Bjørkli T Eldridge S Buchbinder R Underwood M Froud R. Inclusion and exclusion criteria used in non-specific low back pain trials: a review of randomised controlled trials published between 2006 and 2012. BMC Musculoskelet Disord 2018;19:113. 10.1186/s12891-018-2034-6 PMC5898037 29650015

[7] Andermann A. Taking action on the social determinants of health in clinical practice: a framework for health professionals. CMAJ 2016;188:E474–E483. 10.1503/cmaj.160177 PMC5135524 27503870

[8] Argentieri MA Amin N Nevado-Holgado AJ Sproviero W Collister JA Keestra SM Kuilman MM Ginos BNR Ghanbari M Doherty A Hunter DJ Alvergne A van Duijn CM. Integrating the environmental and genetic architectures of aging and mortality. Nat Med 2025;31:1016–25. 10.1038/s41591-024-03483-9 PMC11922759 39972219

[9] Arroyave Caicedo NM Parry E Arslan N Park S. Integration of social determinants of health information within the primary care electronic health record: a systematic review of patient perspectives and experiences. BJGP Open 2024;8:BJGPO.2023.0155. 10.3399/BJGPO.2023.0155 PMC11169979 37673433

[10] Berger E Castagné R Chadeau-Hyam M Bochud M d'Errico A Gandini M Karimi M Kivimäki M Krogh V Marmot M Panico S Preisig M Ricceri F Sacerdote C Steptoe A Stringhini S Tumino R Vineis P Delpierre C Kelly-Irving M. Multi-cohort study identifies social determinants of systemic inflammation over the life course. Nat Commun 2019;10:773. 10.1038/s41467-019-08732-x PMC6377676 30770820

[11] Bernstetter A Brown NH Fredhoff B Rhon DI Cook C. Reporting and incorporation of social risks in low back pain and exercise studies: a scoping review. Musculoskelet Sci Pract 2025;77:103310. 10.1016/j.msksp.2025.103310 40127512

[12] Bloch G Rozmovits L Giambrone B. Barriers to primary care responsiveness to poverty as a risk factor for health. BMC Fam Pract 2011;12:62. 10.1186/1471-2296-12-62 PMC3135547 21714925

[13] Brady B Saberi G Santalucia Y Gorgees P Nguyen TT Le H Sidhu B. “Without support CALD patients will be left behind”: a mixed-methods exploration of culturally and linguistically diverse (CALD) client perspectives of telehealth and those of their healthcare providers. J Telemed Telecare 2024;30:1493–506. 10.1177/1357633X231154943 36798034

[14] Brady B Veljanova I Chipchase L. The intersections of chronic noncancer pain: culturally diverse perspectives on disease burden. Pain Med 2019;20:434–45. 10.1093/pm/pny088 29846709

[15] Braveman P Egerter S Williams DR. The social determinants of health: coming of age. Annu Rev Public Health 2011;32:381–98. 10.1146/annurev-publhealth-031210-101218 21091195

[16] Braveman P Gottlieb L. The social determinants of health: it's time to consider the causes of the causes. Public Health Rep 2014;129:19–31. 10.1177/00333549141291S206 PMC3863696 24385661

[17] Braybrook D Bristowe K Timmins L Roach A Day E Clift P Rose R Marshall S Johnson K Sleeman KE Harding R. Communication about sexual orientation and gender between Clinicians, LGBT+ people facing serious illness and their significant others: a qualitative interview study of experiences, preferences and recommendations. BMJ Qual Saf 2023;32:109–20. 10.1136/bmjqs-2022-014792 PMC9887369 36657773

[18] Brcic V Eberdt C Kaczorowski J. Development of a tool to identify poverty in a family practice setting: a pilot study. Int J Fam Med 2011;2011:1–7. 10.1155/2011/812182 PMC3268233 22312547

[19] Briggs AM Jordan JE Sharma S Young JJ Chua J Foster HE Haq SA Huckel Schneider C Jain A Joshipura M Kalla AA Kopansky-Giles D March L Reis FJJ Reyes KAV Soriano ER Slater H. Context and priorities for health systems strengthening for pain and disability in low- and middle-income countries: a secondary qualitative study and content analysis of health policies. Health Policy Plan 2023;38:129–49. 10.1093/heapol/czac061 PMC9923377 35876078

[20] Browne AJ Varcoe C Ford-Gilboe M Nadine Wathen C Smye V Jackson BE Wallace B Pauly BB Herbert CP Lavoie JG Wong ST Blanchet Garneau A. Disruption as opportunity: impacts of an organizational health equity intervention in primary care clinics. Int J Equity Health 2018;17:154. 10.1186/s12939-018-0820-2 PMC6161402 30261924

[21] Browne AJ Varcoe C Ford-Gilboe M Wathen CN; EQUIP Research Team. EQUIP healthcare: an overview of a multi-component intervention to enhance equity-oriented care in primary health care settings. Int J Equity Health 2015;14:152. 10.1186/s12939-015-0271-y PMC4688920 26694168

[22] Browne AJ Varcoe C Ford-Gilboe M Wathen CN Wilson E Bungay V Perrin N. Using a health equity lens to measure patient experiences of care in diverse health care settings. PLoS One 2024;19:e0297721. 10.1371/journal.pone.0297721 PMC11156339 38843218

[23] Buchbinder R van Tulder M Öberg B Costa LM Woolf A Schoene M Croft P Buchbinder R Hartvigsen J Cherkin D Foster NE Maher CG Underwood M van Tulder M Anema JR Chou R Cohen SP Menezes Costa L Croft P Ferreira M Ferreira PH Fritz JM Genevay S Gross DP Hancock MJ Hoy D Karppinen J Koes BW Kongsted A Louw Q Öberg B Peul WC Pransky G Schoene M Sieper J Smeets RJ Turner JA Woolf A. Low back pain: a call for action. Lancet 2018;391:2384–8. 10.1016/S0140-6736(18)30488-4 29573871

[24] Canadian Medical Association. Physicians and health equity: opportunities in practice, 2013.

[25] Canadian Medical Association. Physicians and health equity: opportunities in practice|National Collaborating Centre for Determinants of Health, 2013. Available at: https://nccdh.ca/resources/entry/physicians-and-health-equity. Accessed June 4, 2025.

[26] Canadian Psychological Association. Canadian code of ethics for psychologists. Ontario, Canada: Canadian Psychological Association, 2017 Available at: https://cpa.ca/docs/File/Ethics/CPA_Code_2017_4thEd.pdf.

[27] Cardosa MS. Promoting multidisciplinary pain management in low- and middle-income countries—challenges and achievements. PAIN 2024;165:S39–S49. 10.1097/j.pain.0000000000003369 39560414

[28] Cené CW Peek ME Jacobs E Horowitz CR. Community-based teaching about health disparities: combining education, scholarship, and community service. J Gen Intern Med 2010;25:S130–S135. 10.1007/s11606-009-1214-3 PMC2847108 20352507

[29] Centers for Medicare and Medicaid Services. A guide to using the accountable health communities health-related social needs screening tool: promising practices and key insights, 2023. Available at: https://www.cms.gov/priorities/innovation/media/document/ahcm-screeningtool-companion.

[30] Chalmers I Glasziou P. Avoidable waste in the production and reporting of research evidence. Lancet 2009;374:86–9. 10.1016/S0140-6736(09)60329-9 19525005

[31] Chambers CT Dol J Tutelman PR Langley CL Parker JA Cormier BT Macfarlane GJ Jones GT Chapman D Proudfoot N Grant A Marianayagam J. The prevalence of chronic pain in children and adolescents: a systematic review update and meta-analysis. PAIN 2024;165:2215–34. 10.1097/j.pain.0000000000003267 PMC11404345 38743558

[32] Reading C Wien F. Health inequalities and social determinants of Aboriginal peoples' health. Price George, BC: National Collaborating Centre for Aboriginal Health, 2009. Available at: https://www.ccnsa-nccah.ca/docs/determinants/RPT-HealthInequalities-Reading-Wien-EN.pdf.

[33] Chatterjee HJ Camic PM Lockyer B Thomson LJM. Non-clinical community interventions: a systematised review of social prescribing schemes. Arts Health 2018;10:97–123.

[34] McGill University. CLEAR collaboration. Montreal, Canada: CLEAR Collaboration, 2025. Available at: https://www.mcgill.ca/clear/. Accessed June 5, 2025.

[35] Comachio J Purcell K Brady B Thiveos T Strkljevic I Shu C Carvalho-E-Silva AP Nikpour M Anderson DB, Improving musculoskeletal health by incorporating equity, diversity, and inclusion approaches into research practices. Musculoskeletal Care 2024;22:e1943. 10.1002/msc.1943 39349352

[36] Commission of Social Determinants of Health. Closing the gap in a generation: Health equity through action on the social determinants of health. Geneva, Switzerland: World Health Organization, 2008. 10.1016/S0140-6736(08)61690-6 18994664

[37] Connoy L Solomon M Longo R Sud A Katz J Dale C Stanley M Webster F. Attending to marginalization in the chronic pain literature: a scoping review. Can J Pain 2024;8:2335500. 10.1080/24740527.2024.2335500 PMC11146439 38831969

[38] Connoy L Webster F. Why language matters in chronic pain: the example of pain catastrophizing. J Pain 2024;25:588–90. 10.1016/j.jpain.2023.12.012 38159787

[39] NHMRC. Consumer and community engagement, 2025. Available at: https://www.nhmrc.gov.au/about-us/consumer-and-community-involvement/consumer-and-community-engagement. Accessed June 4, 2025.

[40] Cordero DM Miclau TA Paul AV Morshed S Miclau T Martin C Shearer DW. The global burden of musculoskeletal injury in low and lower-middle income countries: a systematic literature review. OTA Int 2020;3:e062. 10.1097/OI9.0000000000000062 PMC8022900 33937696

[41] Corline A Cole F Trewern L Penlington C. “Power to the people, to the people”: training for social prescribers improves support of persistent pain. Br J Pain 2023;17:281–92. 10.1177/20494637231152979 PMC10278448 37342392

[42] Costa N Schneider CH Amorim A Parambath S Blyth F. “All of these things interact, that's why it's such a wicked problem”: stakeholders' perspectives of what hinders low back pain care in Australia and how to improve it. Health Res Pol Syst 2024;22:151. 10.1186/s12961-024-01222-7 PMC11552357 39529131

[43] Cowell I O'Sullivan P O'Sullivan K Poyton R McGregor A Murtagh G. Perceptions of physiotherapists towards the management of non-specific chronic low back pain from a biopsychosocial perspective: a qualitative study. Musculoskelet Sci Pract 2018;38:113–9. 10.1016/j.msksp.2018.10.006 30423526

[44] Craig KD Holmes C Hudspith M Moor G Moosa-Mitha M Varcoe C Wallace B. Pain in persons who are marginalized by social conditions. PAIN 2020;161:261–5. 10.1097/j.pain.0000000000001719 PMC6970566 31651578

[45] Crameri P Barrett C Latham J Whyte C. It is more than sex and clothes: culturally safe services for older lesbian, gay, bisexual, transgender and intersex people. Australas J Ageing 2015;34:21–5. 10.1111/ajag.12270 26525442

[46] Crawford R. Healthism and the medicalization of everyday life. Int J Health Serv 1980;10:365–88. 10.2190/3H2H-3XJN-3KAY-G9NY 7419309

[47] Crenshaw K. Demarginalizing the intersection of race and sex: a Black feminist critique of antidiscrimination doctrine, feminist theory and antiracist politics. Feminist legal theories. London, United Kingdom: Routledge, 1997.

[48] Crenshaw K. Mapping the margins: intersectionality, identity politics, and violence against women of color. Stanford L Rev 1991;43:1241–99.

[49] Curtis E Jones R Tipene-Leach D Walker C Loring B Paine SJ Reid P. Why cultural safety rather than cultural competency is required to achieve health equity: a literature review and recommended definition. Int J Equity Health 2019;18:174–17. 10.1186/s12939-019-1082-3 PMC6857221 31727076

[50] Dahlhamer J Lucas J Zelaya C Nahin R Mackey S DeBar L Kerns R Von Korff M Porter L Helmick C. Prevalence of chronic pain and high-impact chronic pain among adults—United States, 2016. MMWR Morb Mortal Wkly Rep 2018;67:1001–6. 10.15585/mmwr.mm6736a2 PMC6146950 30212442

[51] Davenport TE Griech SF VanDeCarr T Rethorn ZD Magnusson DM. Social power and the movement system: why and how physical therapists might influence the upstream currents of health. Phys Ther 2023;103:pzad052. 10.1093/ptj/pzad052 37249541

[52] Davies C Devan H Reid S Haribhai-Thompson J Hempel D Aho-White IJT Te Morenga L. “When you’re in pain you do go into your shell” A community-based pain management programme co-designed with Māori whānau to address inequities to pain management—a qualitative case study. J Pain 2024;25:104760. 10.1016/j.jpain.2024.104760 39730021

[53] De Ruddere L Craig KD. Understanding stigma and chronic pain: a-state-of-the-art review. PAIN 2016;157:1607–10. 10.1097/j.pain.0000000000000512 26859821

[54] Determinants of Health for First Nations people. Australian Institute of Health and Welfare, 2024. Available at: https://www.aihw.gov.au/reports/australias-health/social-determinants-and-indigenous-health. Accessed June 5, 2025.

[55] Dewidar O Shamseer L Melendez-Torres GJ Akl EA Ramke J Wang X Oloyede O Young T Nicholls SG Marshall Z Kennedy M Hardy B-J Rizvi A Ghogomu E Rader T Waddington HS Shea B Nkangu M Ellingwood H Wolfenden L Tufte J Horsley T Pottie K Cuervo LG Juando-Prats C Feng C Sharp MK Little J Owusu-Addo E Francis D Kredo T Mahande MJ Chamberlain C Pantoja T von Elm E Bhutta ZA Tugwell P Wiysonge CS Funnell S Jull J Mbuagbaw L Welch V. Improving the reporting on health equity in observational research (STROBE-equity): extension checklist and elaboration. BMJ 2025;390:e083882. 10.1136/bmj-2024-083882 PMC12406149 40903054

[56] Donkin A Goldblatt P Allen J Nathanson V Marmot M. Global action on the social determinants of health. BMJ Glob Health 2018;3:e000603. 10.1136/bmjgh-2017-000603 PMC5759713 29379648

[57] Dunn M Rushton AB Mistry J Soundy A Heneghan NR. The biopsychosocial factors associated with development of chronic musculoskeletal pain. An umbrella review and meta-analysis of observational systematic reviews. PLoS One 2024;19:e0294830. 10.1371/journal.pone.0294830 PMC10984407 38557647

[58] Emerson AJ Einhorn L Groover M Naze G Baxter GD. Clinical conversations in the management of chronic musculoskeletal pain in vulnerable patient populations: a meta-ethnography. Disabil Rehabil 2023;45:3409–34. 10.1080/09638288.2022.2130447 36205554

[59] De Marchis EmiliaH Brown Erika Aceves Benjamín Loomba Vishalli Molina Melanie Cartier Yuri Wing Holly Gottlieb LauraM. State of the science of screening in healthcare settings. San Francisco: SIREN: Social Interventions Research and Evaluation Network, 2022 Available at: https://sirenetwork.ucsf.edu/sites/default/files/2022-06/final%20SCREEN%20State-of-Science-Report%5B55%5D.pdf.

[60] Exercise and Sports Science Australia. Accredited exercise physiologist professinoal standards for accreditation. Exercise and Sports Science Australia, 2021. Available at: https://www.essa.org.au/Common/Uploaded%20files/Standards/AEP%20Professional%20Standards_170921_FINAL.pdf.

[61] Ferreira ML de Luca K Haile LM Steinmetz JD Culbreth GT Cross M Kopec JA Ferreira PH Blyth FM Buchbinder R Hartvigsen J Wu A-M Safiri S Woolf AD Collins GS Ong KL Vollset SE Smith AE Cruz JA Fukutaki KG Abate SM Abbasifard M Abbasi-Kangevari M Abbasi-Kangevari Z Abdelalim A Abedi A Abidi H Adnani QES Ahmadi A Akinyemi RO Alamer AT Alem AZ Alimohamadi Y Alshehri MA Alshehri MM Alzahrani H Amini S Amiri S Amu H Andrei CL Andrei T Antony B Arabloo J Arulappan J Arumugam A Ashraf T Athari SS Awoke N Azadnajafabad S Bärnighausen TW Barrero LH Barrow A Barzegar A Bearne LM Bensenor IM Berhie AY Bhandari BB Bhojaraja VS Bijani A Bodicha BBA Bolla SR Brazo-Sayavera J Briggs AM Cao C Charalampous P Chattu VK Cicuttini FM Clarsen B Cuschieri S Dadras O Dai X Dandona L Dandona R Dehghan A Demie TGG Denova-Gutiérrez E Dewan SMR Dharmaratne SD Dhimal ML Dhimal M Diaz D Didehdar M Digesa LE Diress M Do HT Doan LP Ekholuenetale M Elhadi M Eskandarieh S Faghani S Fares J Fatehizadeh A Fetensa G Filip I Fischer F Franklin RC Ganesan B Gemeda BNB Getachew ME Ghashghaee A Gill TK Golechha M Goleij P Gupta B Hafezi-Nejad N Haj-Mirzaian A Hamal PK Hanif A Harlianto NI Hasani H Hay SI Hebert JJ Heidari G Heidari M Heidari-Soureshjani R Hlongwa MM Hosseini M-S Hsiao AK Iavicoli I Ibitoye SE Ilic IM Ilic MD Islam SMS Janodia MD Jha RP Jindal HA Jonas JB Kabito GG Kandel H Kaur RJ Keshri VR Khader YS Khan EA Khan MJ Khan MA Khayat Kashani HR Khubchandani J Kim YJ Kisa A Klugarová J Kolahi A-A Koohestani HR Koyanagi A Kumar GA Kumar N Lallukka T Lasrado S Lee W-C Lee YH Mahmoodpoor A Malagón-Rojas JN Malekpour M-R Malekzadeh R Malih N Mehndiratta MM Mehrabi Nasab E Menezes RG Mentis A-FA Mesregah MK Miller TR Mirza-Aghazadeh-Attari M Mobarakabadi M Mohammad Y Mohammadi E Mohammed S Mokdad AH Momtazmanesh S Monasta L Moni MA Mostafavi E Murray CJL Nair TS Nazari J Nejadghaderi SA Neupane S Neupane Kandel S Nguyen CT Nowroozi A Okati-Aliabad H Omer E Oulhaj A Owolabi MO Panda-Jonas S Pandey A Park E-K Pawar S Pedersini P Pereira J Peres MFP Petcu I-R Pourahmadi M Radfar A Rahimi-Dehgolan S Rahimi-Movaghar V Rahman M Rahmani AM Rajai N Rao CR Rashedi V Rashidi M-M Ratan ZA Rawaf DL Rawaf S Renzaho AMN Rezaei N Rezaei Z Roever L Ruela GdA Saddik B Sahebkar A Salehi S Sanmarchi F Sepanlou SG Shahabi S Shahrokhi S Shaker E Shamsi M Shannawaz M Sharma S Shaygan M Sheikhi RA Shetty JK Shiri R Shivalli S Shobeiri P Sibhat MM Singh A Singh JA Slater H Solmi M Somayaji R Tan K-K Thapar R Tohidast SA Valadan Tahbaz S Valizadeh R Vasankari TJ Venketasubramanian N Vlassov V Vo B Wang Y-P Wiangkham T Yadav L Yadollahpour A Yahyazadeh Jabbari SH Yang L Yazdanpanah F Yonemoto N Younis MZ Zare I Zarrintan A Zoladl M Vos T March LM. Global, regional, and national burden of low back pain, 1990–2020, its attributable risk factors, and projections to 2050: a systematic analysis of the Global Burden of Disease Study 2021. Lancet Rheumatol 2023;5:e316–e329. 10.1016/S2665-9913(23)00098-X PMC10234592 37273833

[62] FitzGerald C Hurst S. Implicit bias in healthcare professionals: a systematic review. BMC Med Ethics 2017;18:19. 10.1186/s12910-017-0179-8 PMC5333436 28249596

[63] Ford-Gilboe M Wathen CN Varcoe C Herbert C Jackson BE Lavoie JG Pauly B Perrin NA Smye V Wallace B Wong ST; BROWNE for the EQUIP Research Program AJ. How equity-oriented health care affects health: key mechanisms and implications for primary health care practice and Policy. Milbank Q 2018;96:635–71. 10.1111/1468-0009.12349 PMC6287068 30350420

[64] Garg A Boynton-Jarrett R Dworkin PH. Avoiding the unintended consequences of screening for social determinants of health. JAMA 2016;316:813–4. 10.1001/jama.2016.9282 27367226

[65] Gaspar Fernandes L Davies C Jaye C Hay-Smith J Devan H. “We do not stop being Indigenous when we are in pain”: an integrative review of the lived experiences of chronic pain among Indigenous peoples. Soc Sci Med 2025;373:117991. 10.1016/j.socscimed.2025.117991 40158447

[66] Wu AM Cross, M Elliott, JM Culbreth, GT Haile, LM Steinmetz, JD Hagins, H Kopec, JA Brooks, PM Woolf, AD Kopansky-Giles, DR Walton, DM Treleaven, JM Dreinhoefer, KE Betteridge, N Abbasifard, M Abbasi-Kangevari, Z Addo, IY Adesina, MA Adnani, QES Aithala, JP Alhalaiqa, FAN Alimohamadi, Y Amiri, S Amu, H Antony, B Arabloo, J Aravkin, AY Asghari-Jafarabadi, M Atomsa, GH Azadnajafabad, S Azzam, AY Baghdadi, S Balogun, SA Balta, AB Banach, M Banakar, M Barrow, A Bashiri, A Bekele, A Bensenor, IM Bhardwaj, P Bhat, AN Bilchut, AH Briggs, AM Buchbinder, R Cao, C Chaurasia, A Chirinos-Caceres, JL Christensen, SWM Coberly, K Cousin, E Dadras, O Dai, X de Luca, K Dehghan, A Dong, HJ Ekholuenetale, M Elhadi, M Eshetu, HB Eskandarieh, S Etaee, F Fagbamigbe, AF Fares, J Fatehizadeh, A Feizkhah, A Ferreira, ML Ferreira, N Fischer, F Franklin, RC Ganesan, B Gebremichael, MA Gerema, U Gholami, A Ghozy, S Gill, TK Golechha, M Goleij, P Golinelli, D Graham, SM Haj-Mirzaian, A Harlianto, NI Hartvigsen, J Hasanian, M Hassen, MB Hay, SI Hebert, JJ Heidari, G Hoveidaei, AH Hsiao, AK Ibitoye, SE Iwu, CCD Jacob, L Janodia, MD Jin, Y Jonas, JB Joshua, CE Kandel, H Khader, YS Khajuria, H Khan, EA Khan, MA Khatatbeh, MM Khateri, S Khayat Kashani, HR Khonji, MS Khubchandani, J Kim, YJ Kisa, A Kolahi, AA Koohestani, HR Krishan, K Kuddus, M Kuttikkattu, A Lasrado, S Lee, YH Legesse, SM Lim, SS Liu, X Lo, J Malih, N Manandhar, SP Mathews, E Mesregah, MK Mestrovic, T Miller, TR Mirghaderi, SP Misganaw, A Mohammadi, E Mohammed, S Mokdad, AH Momtazmanesh, S Moni, MA Mostafavi, E Murray, CJL Nair, TS Nejadghaderi, SA Nzoputam, OJ Oh, IH Okonji, OC Owolabi, MO Pacheco-Barrios, K Pahlevan Fallahy, MT Park, S Patel, J Pawar, S Pedersini, P Peres, MFP Petcu, IR Pourahmadi, M Qattea, I Ram, P Rashidi, MM Rawaf, S Rezaei, N Rezaei, N Saeed, U Saheb Sharif-Askari, F Salahi, S Sawhney, M Schumacher, AE Shafie, M Shahabi, S Shahbandi, A Shamekh, A Sharma, S Shiri, R Shobeiri, P Sinaei, E Singh, A Singh, JA Singh, P Skryabina, AA Smith, AE Tabish, M Tan, KK Tegegne, MD Tharwat, S Vahabi, SM Valadan Tahbaz, S Vasankari, TJ Venketasubramanian, N Vollset, SE Wang, YP Wiangkham, T Yonemoto, N Zangiabadian, M Zare, I Zemedikun, DT Zheng, P Ong, KL Vos, T March, LM. Global, regional, and national burden of neck pain, 1990-2020, and projections to 2050: a systematic analysis of the Global Burden of Disease Study 2021. Lancet Rheumatol 2024;6:e142–e155. 10.1016/S2665-9913(23)00321-1 PMC10897950 38383088

[67] Geneva: World Health Organization. World report on social determinants of health equity. Geneva: WHO, 2025. Available at: https://www.who.int/teams/social-determinants-of-health/equity-and-health/world-report-on-social-determinants-of-health-equity.

[68] Gibbs MT Morrison NMV Marshall PWM. Biomedical beliefs explain the clinical decisions made by exercise-based practitioners for people with chronic low back pain. Spine 2021;46:114–21. 10.1097/BRS.0000000000003698 32947498

[69] Glare P Overton S Aubrey K. Transition from acute to chronic pain: where cells, systems and society meet. Pain Manag 2020;10:421–36. 10.2217/pmt-2019-0039 33111634

[70] Gottlieb LM Hessler D Wing H Gonzalez-Rocha A Cartier Y Fichtenberg C. Revising the logic model behind health care's social care investments. Milbank Q 2024;102:325–35. 10.1111/1468-0009.12690 PMC11176407 38273221

[71] Goucke CR Chaudakshetrin P. Pain: a neglected problem in the low-resource setting. Anesth Analgesia 2018;126:1283–6. 10.1213/ANE.0000000000002736 29547421

[72] Government of Canada CI of HR. Strategy for patient-oriented research, 2018. Available at: https://cihr-irsc.gc.ca/e/41204.html. Accessed June 4, 2025.

[73] Green CR Anderson KO Baker TA Campbell LC Decker S Fillingim RB Kaloukalani DA Lasch KE Myers C Tait RC Todd KH Vallerand AH. The unequal burden of pain: confronting racial and ethnic disparities in pain. Pain Med 2003;4:277–94. 10.1046/j.1526-4637.2003.03034.x 12974827

[74] Harrison LE Boerner KE Black W Nelson S Santos M Simons LE Wakefield EO Warner JN Wilson AC Zajacova A. A call to action to integrate best practice assessment of sexual orientation and gender identity in pain research and clinical care. PAIN 2025;166:988–93. 10.1097/j.pain.0000000000003481 39679643

[75] Hartvigsen J Hancock MJ Kongsted A Louw Q Ferreira ML Genevay S Hoy D Karppinen J Pransky G Sieper J Smeets RJ Underwood M Buchbinder R Hartvigsen J Cherkin D Foster NE Maher CG Underwood M van Tulder M Anema JR Chou R Cohen SP Menezes Costa L Croft P Ferreira M Ferreira PH Fritz JM Genevay S Gross DP Hancock MJ Hoy D Karppinen J Koes BW Kongsted A Louw Q Öberg B Peul WC Pransky G Schoene M Sieper J Smeets RJ Turner JA Woolf A. What low back pain is and why we need to pay attention. Lancet 2018;391:2356–67. 10.1016/S0140-6736(18)30480-X 29573870

[76] Health professionals council of South Africa—psychology, 2025. Available at: https://www.hpcsa.co.za/board/psychology. Accessed June 5, 2025.

[77] Heard E Fitzgerald L Wigginton B Mutch A. Applying intersectionality theory in health promotion research and practice. Health Promot Int 2020;35:866–76. 10.1093/heapro/daz080 31390472

[78] van Hecke O Torrance N Smith BH. Chronic pain epidemiology and its clinical relevance. Br J Anaesth 2013;111:13–8. 10.1093/bja/aet123 23794640

[79] Henning M Smith M. The ability of physiotherapists to identify psychosocial factors in patients with musculoskeletal pain: a scoping review. Musculoskeletal Care 2023;21:502–15. 10.1002/msc.1725 36564962

[80] Hiam L Klaber B Sowemimo A Marmot M. NHS and the whole of society must act on social determinants of health for a healthier future. BMJ 2024;385:e079389. 10.1136/bmj-2024-079389 38604669

[81] HIAT—FOR-EQUITY. Available at: https://forequity.uk/hiat/. Accessed 5 June 2025.

[82] Home: Risk screening tools review. Available at: https://sdh-tools-review.kpwashingtonresearch.org/. Accessed June 5, 2025.

[83] Hood CM Gennuso KP Swain GR Catlin BB. County health rankings: relationships between determinant factors and health outcomes. Am J Prev Med 2016;50:129–35. 10.1016/j.amepre.2015.08.024 26526164

[84] van Griensven H Strong J. Pain: a textbook for health professionals. 3rd ed. Geneva: Elsevier, 2023.

[85] Hudon C Loignon C Grabovschi C Bush P Lambert M Goulet É Boyer S De Laat M Fournier N. Medical education for equity in health: a participatory action research involving persons living in poverty and healthcare professionals. BMC Med Educ 2016;16:106. 10.1186/s12909-016-0630-4 PMC4828813 27066826

[86] Hughes S Pincus T Geraghty A Chew-Graham C Stuart B Little P Moore M Birkinshaw H. Planning and developing an intervention to improve the psychological wellbeing of people living with persistent pain: de-stress pain. PAIN 2024.

[87] Jackson T Thomas S Stabile V Shotwell M Han X McQueen K. A systematic review and meta-analysis of the global burden of chronic pain without clear etiology in low- and middle-income countries: trends in heterogeneous data and a proposal for new assessment methods. Anesth Analgesia 2016;123:739–48. 10.1213/ANE.0000000000001389 27537761

[88] Janevic MR Mathur VA Booker SQ Morais C Meints SM Yeager KA Meghani SH. Making pain research more inclusive: why and how. J Pain 2022;23:707–28. 10.1016/j.jpain.2021.10.004 PMC9018873 34678471

[89] Jimenez N Garroutte E Kundu A Morales L Buchwald D. A review of the experience, epidemiology, and management of pain among American Indian, Alaska Native, and Aboriginal Canadian peoples. J Pain 2011;12:511–22. 10.1016/j.jpain.2010.12.002 PMC3090505 21330217

[90] Johnson MI Page K Woodall J Thompson K. Perspectives on community-based system change for people living with persistent pain: insights from developing the “Rethinking Pain Service” . Front Pain Res (Lausanne) 2024;5:1299027. 10.3389/fpain.2024.1299027 PMC10987759 38571563

[91] Kamper SJ Apeldoorn AT Chiarotto A Smeets RJ Ostelo RW Guzman J van Tulder MW. Multidisciplinary biopsychosocial rehabilitation for chronic low back pain. Cochrane Database Syst Rev 2014;2014:CD000963. 10.1002/14651858.CD000963.pub3 PMC10945502 25180773

[92] Kapos FP Craig KD Anderson SR Bernardes SF Hirsh AT Karos K Keogh E Reynolds Losin EA McParland JL Moore DJ Ashton-James CE. Social determinants and consequences of pain: toward multilevel, intersectional, and life course perspectives. J Pain 2024;25:104608. 10.1016/j.jpain.2024.104608 PMC11402600 38897311

[93] Karran EL Cashin AG Barker T Boyd MA Chiarotto A Dewidar O Mohabir V Petkovic J Sharma S Tejani S Tugwell P Moseley GL. Using PROGRESS-plus to identify current approaches to the collection and reporting of equity-relevant data: a scoping review. J Clin Epidemiol 2023;163:70–8. 10.1016/j.jclinepi.2023.09.017 37802205

[94] Karran EL G Cashin A Barker T A Boyd M Chiarotto A Dewidar O Petkovic J Sharma S Tugwell P Moseley GL. The “what” and “how” of screening for social needs in healthcare settings: a scoping review. PeerJ 2023;11:e15263. 10.7717/peerj.15263 PMC10124546 37101795

[95] Karran EL Cashin AG Barker T Boyd MA Chiarotto A Maxwell LJ Mohabir V Sharma S Tugwell P Moseley GL. Developing consensus on the most important equity-relevant items to include in pain research: a modified e-delphi study. PAIN 2025;166:e378–e387. 10.1097/j.pain.0000000000003621 40258130

[96] Karran EL Cashin AG Barker T Boyd MA Chiarotto A Mohabir V Petkovic J Sharma S Tugwell P Moseley GL. It is time to take a broader equity lens to highlight health inequalities in people with pain. Br J Anaesth 2025;134:235–7. 10.1016/j.bja.2024.09.026 39505590

[97] Karran EL Grant AR Moseley GL. Low back pain and the social determinants of health: a systematic review and narrative synthesis. PAIN 2020;161:2476–93. 10.1097/j.pain.0000000000001944 32910100

[98] Kazi S Starling C Milicia A Buckley B Grisham R Gruber E Miller K Arem H. Barriers and facilitators to screen for and address social needs in primary care practices in Maryland: a qualitative study. Front Health Serv 2024;4:1380589. 10.3389/frhs.2024.1380589 PMC11215188 38952646

[99] Kohrt BA Griffith JL Patel V. Chronic pain and mental health: integrated solutions for global problems. PAIN 2018;159:S85–S90. 10.1097/j.pain.0000000000001296 PMC6130207 30113952

[100] Kostelanetz S Pettapiece-Phillips M Weems J Spalding T Roumie C Wilkins CH Kripalani S. Health care professionals' perspectives on universal screening of social determinants of health: a mixed-methods study. Popul Health Manage 2022;25:367–74. 10.1089/pop.2021.0176 PMC11913136 34698559

[101] Kuntz B Hoebel J Fuchs J Neuhauser H Lampert T. Social inequalities in the prevalence of chronic back pain among adults in Germany. Bundesgesundheitsblatt Gesundheitsforschung Gesundheitsschutz 2017;60:783–91. 10.1007/s00103-017-2568-z 28516263

[102] Lacey RJ Belcher J Croft PR. Does life course socio-economic position influence chronic disabling pain in older adults? A general population study. Eur J Public Health 2013;23:534–40. 10.1093/eurpub/cks056 PMC3719471 22874735

[103] Latimer M Rudderham S Lethbridge L MacLeod E Harman K Sylliboy JR Filiaggi C Finley GA. Occurrence of and referral to specialists for pain-related diagnoses in first nations and non-first nations children and youth. CMAJ 2018;190:E1434–E1440. 10.1503/cmaj.180198 PMC6279448 30530610

[104] Letzen JE Mathur VA Janevic MR Burton MD Hood AM Morais CA Booker SQ Campbell CM Aroke EN Goodin BR Campbell LC Merriwether EN. Confronting racism in all forms of pain research: reframing study designs. J Pain 2022;23:893–912. 10.1016/j.jpain.2022.01.010 PMC9472383 35296390

[105] Lin I Coffin J Bullen J Barnabe C. Opportunities and challenges for physical rehabilitation with indigenous populations. PAIN Rep 2020;5:e838. 10.1097/PR9.0000000000000838 PMC7808686 33490838

[106] Lin IB Bunzli S Mak DB Green C Goucke R Coffin J O'Sullivan PB. Unmet needs of Aboriginal Australians with musculoskeletal pain: a mixed-method systematic review. Arthritis Care Res 2018;70:1335–1347. 10.1002/acr.23493 29245188

[107] Livingston CJ Titus TM Yerokun TA Patel NA. Screening for health-related social needs: american college of preventive medicine's practice statement. Am J Prev Med 2025;68:1041–9. 10.1016/j.amepre.2025.01.005 39793769

[108] Gottlieb L Fichtenberg C Adler N. Introducing the social interventions research and evaluation network. San Francisco, CA: SIREN, 2017. Available at: https://sirenetwork.ucsf.edu/sites/default/files/SIREN_Issue_Brief_Updt.pdf.

[109] Luchenski S Maguire N Aldridge RW Hayward A Story A Perri P Withers J Clint S Fitzpatrick S Hewett N. What works in inclusion health: overview of effective interventions for marginalised and excluded populations. Lancet 2018;391:266–80. 10.1016/S0140-6736(17)31959-1 29137868

[110] Lucyk K McLaren L. Taking stock of the social determinants of health: a scoping review. PLoS One 2017;12:e0177306. 10.1371/journal.pone.0177306 PMC5426664 28493934

[111] Lynch ME Campbell F Clark AJ Dunbar MJ Goldstein D Peng P Stinson J Tupper H. A systematic review of the effect of waiting for treatment for chronic pain. PAIN 2008;136:97–116. 10.1016/j.pain.2007.06.018 17707589

[112] Macchia L Oswald AJ. Physical pain, gender, and the state of the economy in 146 nations. Soc Sci Med 2021;287:114332. 10.1016/j.socscimed.2021.114332 34500321

[113] Macgregor C Walumbe J Tulle E Seenan C Blane DN. Intersectionality as a theoretical framework for researching health inequities in chronic pain. Br J Pain 2023;17:479–90. 10.1177/20494637231188583 PMC10722103 38107758

[114] MacNeela P Doyle C O'Gorman D Ruane N McGuire BE. Experiences of chronic low back pain: a meta-ethnography of qualitative research. Health Psychol Rev 2015;9:63–82. 10.1080/17437199.2013.840951 25793491

[115] Magnan S. Social determinants of health 201 for health care: plan, do, study, act. NAM Perspect 2021;2021:10. 10.31478/202106c PMC8406598 34532697

[116] Marmot M. Achieving health equity: from root causes to fair outcomes. Lancet 2007;370:1153–63. 10.1016/S0140-6736(07)61385-3 17905168

[117] Marmot M. Social determinants of health inequalities. Lancet 2005;365:1099–104. 10.1016/S0140-6736(05)71146-6 15781105

[118] Marmot M. Social justice, epidemiology and health inequalities. Eur J Epidemiol 2017;32:537–46. 10.1007/s10654-017-0286-3 PMC5570780 28776115

[119] McCartney G Popham F McMaster R Cumbers A. Defining health and health inequalities. Public Health 2019;172:22–30. 10.1016/j.puhe.2019.03.023 PMC6558275 31154234

[120] McKivett A Paul D Hudson N. Healing conversations: developing a practical framework for clinical communication between Aboriginal communities and healthcare practitioners. J Immigrant Minor Health 2019;21:596–605. 10.1007/s10903-018-0793-7 30066058

[121] McNeil R Guirguis-Younger M Dilley LB Turnbull J Hwang SW. Learning to account for the social determinants of health affecting homeless persons. Med Educ 2013;47:485–94. 10.1111/medu.12132 23574061

[122] Meints SM Cortes A Morais CA Edwards RR. Racial and ethnic differences in the experience and treatment of noncancer pain. Pain Manag 2019;9:317–34. 10.2217/pmt-2018-0030 PMC6587104 31140916

[123] Mescouto K Olson RE Hodges PW Costa N Patton MA Evans K Walsh K Lonergan K Setchell J. Physiotherapists both reproduce and resist biomedical dominance when working with people with low back pain: a qualitative Study towards new praxis. Qual Health Res 2022;32:902–15. 10.1177/10497323221084358 35341400

[124] Mescouto K Olson RE Hodges PW Setchell J. A critical review of the biopsychosocial model of low back pain care: time for a new approach? Disabil Rehabil 2022;44:3270–84. 10.1080/09638288.2020.1851783 33284644

[125] Marmot M Allen J Goldblatt P Boyce T McNeish D Grady M. Fair society, healthy lives: the marmot review. Institute of Health Equity, 2010 Available at: https://www.instituteofhealthequity.org/resources-reports/fair-society-healthy-lives-the-marmot-review/fair-society-healthy-lives-full-report-pdf.pdf.

[126] Marmot M Allen J Boyce T Goldblatt P Morrison J. Health equity in England: The marmot review 10 years on. Institute of health equity, 2020. Available at: https://www.instituteofhealthequity.org/resources-reports/marmot-review-10-years-on/the-marmot-review-10-years-on-full-report.pdf. 10.1136/bmj.m693 32094110

[127] Mills SEE Nicolson KP Smith BH. Chronic pain: a review of its epidemiology and associated factors in population-based studies. Br J Anaesth 2019;123:e273–e283. 10.1016/j.bja.2019.03.023 PMC6676152 31079836

[128] Mojdehi S Brady B Tang C Peiris CL. The effectiveness of multidisciplinary, activity-based chronic pain interventions for adults of ethnoculturally diverse backgrounds: a systematic review with meta-analysis. Disabil Rehabil 2025;47:314–23. 10.1080/09638288.2024.2349761 38720522

[129] Moore GF Audrey S Barker M Bond L Bonell C Hardeman W Moore L O'Cathain A Tinati T Wight D Baird J. Process evaluation of complex interventions: medical research council guidance. BMJ 2015;350:h1258. 10.1136/bmj.h1258 PMC4366184 25791983

[130] Morris LD Daniels KJ Ganguli B Louw QA. An update on the prevalence of low back pain in Africa: a systematic review and meta-analyses. BMC Musculoskelet Disord 2018;19:196. 10.1186/s12891-018-2075-x PMC6055346 30037323

[131] National Academies of Sciences, Engineering, and Medicine; Health and Medicine Division; Board on Health Care Services; Committee on Integrating Social Needs Care into the Delivery of Health Care to Improve the Nation's Health. Integrating social care into the delivery of health care: moving upstream to improve the nation's health. Washington (DC): National Academies Press (US), 2019. Available at: http://www.ncbi.nlm.nih.gov/books/NBK552597/. Accessed June 5, 2025.

[132] National Academies of Sciences, Engineering, and Medicine; National Academy of Medicine; Committee on the Future of Nursing 2020–2030. Flaubert JL Le Menestrel S Williams DR Wakefield MK, editors. The Future of Nursing 2020–2030: Charting a Path to Achieve Health Equity. Washington (DC): National Academies Press (US), 2021. Available at: http://www.ncbi.nlm.nih.gov/books/NBK573914/. Accessed June 4, 2025. 34524769

[133] National Pain Advocacy Center. National pain advocacy center. Available at: https://nationalpain.org. Accessed June 5, 2025.

[134] Naz A Rosenberg E Andersson N Labonté R Andermann A; CLEAR Collaboration. Health workers who ask about social determinants of health are more likely to report helping patients: mixed-methods study. Can Fam Physician 2016;62:e684–e693. PMC9844577 28661888

[135] Ng W Slater H Starcevich C Wright A Mitchell T Beales D. Barriers and enablers influencing healthcare professionals' adoption of a biopsychosocial approach to musculoskeletal pain: a systematic review and qualitative evidence synthesis. PAIN 2021;162:2154–85. 10.1097/j.pain.0000000000002217 33534357

[136] Ocloo J Matthews R. From tokenism to empowerment: progressing patient and public involvement in healthcare improvement. BMJ Qual Saf 2016;25:626–32. 10.1136/bmjqs-2015-004839 PMC4975844 26993640

[137] Solar O Irwin A. A conceptual framework for action on the social determinants of health. Social determinants of health discussion paper 2 (policy and practice). Geneva: World Health Organization, 2010.

[138] O'Sullivan PB Caneiro JP O'Keeffe M Smith A Dankaerts W Fersum K O'Sullivan K. Cognitive functional therapy: an integrated behavioral approach for the targeted management of disabling low back pain. Phys Ther 2018;98:408–23. 10.1093/ptj/pzy022 PMC6037069 29669082

[139] Ottersen OP Dasgupta J Blouin C Buss P Chongsuvivatwong V Frenk J Fukuda-Parr S Gawanas BP Giacaman R Gyapong J Leaning J Marmot M McNeill D Mongella GI Moyo N Møgedal S Ntsaluba A Ooms G Bjertness E Lie AL Moon S Roalkvam S Sandberg KI Scheel IB. The political origins of health inequity: prospects for change. Lancet 2014;383:630–67. 10.1016/S0140-6736(13)62407-1 24524782

[140] Palermo TM. Editorial: introducing new reporting guidelines to address inclusion, diversity, equity, antiracism, and accessibility: implementation at the journal of pain. J Pain 2023;24:22–3. 10.1016/j.jpain.2022.11.001 36437220

[141] Paradies Y. Racism and indigenous health, 2018.

[142] Pilkington G Johnson MI Thompson K. Social prescribing for adults with chronic pain in the U.K.: a rapid review. Br J Pain 2025;10:20494637241312064. 10.1177/20494637241312064 PMC11752153 39850272

[143] Reis FJJ Nijs J Parker R Sharma S Wideman TH. Culture and musculoskeletal pain: strategies, challenges, and future directions to develop culturally sensitive physical therapy care. Braz J Phys Ther 2022;26:100442. 10.1016/j.bjpt.2022.100442 PMC9550611 36209626

[144] Rethorn ZD Cook C Reneker JC. Social determinants of health: if you aren't measuring them, you aren't seeing the big picture. J Orthopaedic Sports Phys Ther 2019;49:872–4. 10.2519/jospt.2019.0613 31789121

[145] Rinaudo CM Van de Velde M Steyaert A Mouraux A. Navigating the biopsychosocial landscape: a systematic review on the association between social support and chronic pain. PLoS One 2025;20:e0321750. 10.1371/journal.pone.0321750 PMC12040255 40300000

[146] Ritchie CS Patel K Boscardin J Miaskowski C Vranceanu A-M Whitlock E Smith A. Impact of persistent pain on function, cognition, and well-being of older adults. J Am Geriatr Soc 2023;71:26–35. 10.1111/jgs.18125 PMC9871006 36475388

[147] Royal Australian College of General Practitioners. Standards for general practices: 5th Edition. Australia: Royal Australian College of General Practitioners, 2023 Available at: https://www.racgp.org.au/getattachment/ece472a7-9a15-4441-b8e5-be892d4ffd77/Standards-for-general-practices-5th-edition.aspx.

[148] RSPH. Driving forward social prescribing: a framework for allied health professionals. Available at: https://www.rsph.org.uk/our-work/resources/ahp-social-prescribing-frameworks.html. Accessed June 4, 2025.

[149] Schram A Carrad A Townsend B Harris P Baum F Rychetnik L Allender S Pescud M Frank N Arthur M Friel S. Doing (and undoing) privilege: evaluating how public policy drives health inequities. Crit Public Health 2025;35:2474078.

[150] Scott KM Koenen KC Aguilar-Gaxiola S Alonso J Angermeyer MC Benjet C Bruffaerts R Caldas-de-Almeida JM de Girolamo G Florescu S Iwata N Levinson D Lim CCW Murphy S Ormel J Posada-Villa J Kessler RC. Associations between lifetime traumatic events and subsequent chronic physical conditions: a cross-national, cross-sectional study. PLoS One 2013;8:e80573. 10.1371/journal.pone.0080573 PMC3864645 24348911

[151] Sharma S Abbott JH Jensen MP. Why clinicians should consider the role of culture in chronic pain. Braz J Phys Ther 2018;22:345–6. 10.1016/j.bjpt.2018.07.002 PMC6157457 30126712

[152] Sharma S Chala MB Resende de Jesus-Moraleida F Than VQ Blyth FM. Tackling the growing burden of pain in low/middle-income countries. PAIN 2025;166:S140–S145. 10.1097/j.pain.0000000000003672 41086346

[153] Sharma S McAuley JH. Low back pain in low- and middle-income countries, part 1: the problem. J Orthopaedic Sports Phys Ther 2022;52:233–235. 10.2519/jospt.2022.11145 35536248

[154] Sharma S Pathak A Jha J Jensen MP. Socioeconomic factors, psychological factors, and function in adults with chronic musculoskeletal pain from rural Nepal. J Pain Res 2018;11:2385–2396. 10.2147/JPR.S173851 PMC6200427 30425551

[155] Sharma S Pathak A Parker R Costa LOP Ghai B Igwesi-Chidobe C Janwantanakul P de Jesus-Moraleida FR Chala MB Pourahmadi M Briggs AM Gorgon E Ardern CL Khan KM McAuley JH Alghwiri A Aoko OA Badamasi HS Calvache JA Cardosa MS Ganesh S Gashaw M Ghiringhelli J Gigena S Hasan AT Haq SA Jacob EN Janse van Rensburg DC Kossi O Liu C Malani R Mason BJN Najem C Nava-Bringas TI Nduwimana I Perera R Perveen W Pierobon A Pinto E Pinto RZ Purwanto F Rahimi MD Reis FJ Siddiq MAB Shrestha D Tamang M Vasanthan TL Viljoen C. How low back pain is managed-A mixed-methods study in 32 countries. Part 2 of low back pain in low- and middle-income countries series. J Orthopaedic Sports Phys Ther 2024;54:560–72. 10.2519/jospt.2024.12406 38602844

[156] Sharma S Traeger AC Reed B Hamilton M O'Connor DA Hoffmann TC Bonner C Buchbinder R Maher CG. Clinician and patient beliefs about diagnostic imaging for low back pain: a systematic qualitative evidence synthesis. BMJ Open 2020;10:e037820. 10.1136/bmjopen-2020-037820 PMC7451538 32830105

[157] Sharma S Verhagen A Elkins M Brismée J-M Fulk GD Taradaj J Steen L Jette A Moore A Stewart A Hoogenboom BJ Söderlund A Harms M Pinto RZ. Research from low-income and middle-income countries will benefit global health and the physiotherapy profession, but it requires support. J Physiother 2024;70:pzad081. 10.1016/j.jphys.2023.08.013 37778960

[158] Standards of proficiency: Physiotherapists. The Health and Care Professions Council. 2023. Available at: https://www.hcpc-uk.org/standards/standards-of-proficiency/physiotherapists/. Accessed June 5, 2025.

[159] Suls J Rothman A. Evolution of the biopsychosocial model: prospects and challenges for health psychology. Health Psychol 2004;23:119–25. 10.1037/0278-6133.23.2.119 15008654

[160] Synnott A O'Keeffe M Bunzli S Dankaerts W O'Sullivan P O'Sullivan K. Physiotherapists may stigmatise or feel unprepared to treat people with low back pain and psychosocial factors that influence recovery: a systematic review. J Physiother 2015;61:68–76. 10.1016/j.jphys.2015.02.016 25812929

[161] Tembo D Hickey G Montenegro C Chandler D Nelson E Porter K Dikomitis L Chambers M Chimbari M Mumba N Beresford P Ekiikina PO Musesengwa R Staniszewska S Coldham T Rennard U. Effective engagement and involvement with community stakeholders in the co-production of global health research. BMJ 2021;372:n178. 10.1136/bmj.n178 PMC7879275 33593805

[162] the Health Innovation Network (HIN). An evaluation of link workers in pain clinics. London: HIN, 2023. Available at: https://healthinnovationnetwork.com/wp-content/uploads/2023/11/Evaluation-of-link-workers-in-pain-clinics_FINAL_.pdf.

[163] UK standards for public involvement—the UK standards. Available at: https://sites.google.com/nihr.ac.uk/pi-standards/standards. Accessed June 4, 2025.

[164] Vinnicombe S Bianchim MS Noyes J. A review of reviews exploring patient and public involvement in population health research and development of tools containing best practice guidance. BMC Public Health 2023;23:1271. 10.1186/s12889-023-15937-9 PMC10311710 37391764

[165] Vos T Barber RM Bell B Bertozzi-Villa A Biryukov S Bolliger I Charlson F Davis A Degenhardt L Dicker D Duan L Erskine H Feigin VL Ferrari AJ Fitzmaurice C Fleming T Graetz N Guinovart C Haagsma J Hansen GM Hanson SW Heuton KR Higashi H Kassebaum N Kyu H Laurie E Liang X Lofgren K Lozano R MacIntyre MF Moradi-Lakeh M Naghavi M Nguyen G Odell S Ortblad K Roberts DA Roth GA Sandar L Serina PT Stanaway JD Steiner C Thomas B Vollset SE Whiteford H Wolock TM Ye P Zhou M Ãvila MA Aasvang GM Abbafati C Ozgoren AA Abd-Allah F Aziz MIA Abera SF Aboyans V Abraham JP Abraham B Abubakar I Abu-Raddad LJ Abu-Rmeileh NM Aburto TC Achoki T Ackerman IN Adelekan A Ademi Z Adou AK Adsuar JC Arnlov J Agardh EE Al Khabouri MJ Alam SS Alasfoor D Albittar MI Alegretti MA Aleman AV Alemu ZA Alfonso-Cristancho R Alhabib S Ali R Alla F Allebeck P Allen PJ AlMazroa MA Alsharif U Alvarez E Alvis-Guzman N Ameli O Amini H Ammar W Anderson BO Anderson HR Antonio CAT Anwari P Apfel H Arsenijevic VSA Artaman A Asghar RJ Assadi R Atkins LS Atkinson C Badawi A Bahit MC Bakfalouni T Balakrishnan K Balalla S Banerjee A Barker-Collo SL Barquera S Barregard L Barrero LH Basu S Basu A Baxter A Beardsley J Bedi N Beghi E Bekele T Bell ML Benjet C Bennett DA Bensenor IM Benzian H Bernabe E Beyene TJ Bhala N Bhalla A Bhutta Z Bienhoff K Bikbov B Abdulhak AB Blore JD Blyth FM Bohensky MA Basara BB Borges G Bornstein NM Bose D Boufous S Bourne RR Boyers LN Brainin M Brauer M Brayne CE Brazinova A Breitborde NJ Brenner H Briggs AD Brooks PM Brown J Brugha TS Buchbinder R Buckle GC Bukhman G Bulloch AG Burch M Burnett R Cardenas R Cabral NL Nonato IRC Campuzano JC Carapetis JR Carpenter DO Caso V Castaneda-Orjuela CA Catala-Lopez F Chadha VK Chang J-C Chen H Chen W Chiang PP Chimed-Ochir O Chowdhury R Christensen H Christophi CA Chugh SS Cirillo M Coggeshall M Cohen A Colistro V Colquhoun SM Contreras AG Cooper LT Cooper C Cooperrider K Coresh J Cortinovis M Criqui MH Crump JA Cuevas-Nasu L Dandona R Dandona L Dansereau E Dantes HG Dargan PI Davey G Davitoiu DV Dayama A De la Cruz-Gongora V de la Vega SF De Leo D del Pozo-Cruz B Dellavalle RP Deribe K Derrett S Des Jarlais DC Dessalegn M deVeber GA Dharmaratne SD Diaz-Torne C Ding EL Dokova K Dorsey ER Driscoll TR Duber H Durrani AM Edmond KM Ellenbogen RG Endres M Ermakov SP Eshrati B Esteghamati A Estep K Fahimi S Farzadfar F Fay DF Felson DT Fereshtehnejad S-M Fernandes JG Ferri CP Flaxman A Foigt N Foreman KJ Fowkes FGR Franklin RC Furst T Futran ND Gabbe BJ Gankpe FG Garcia-Guerra FA Geleijnse JM Gessner BD Gibney KB Gillum RF Ginawi IA Giroud M Giussani G Goenka S Goginashvili K Gona P de Cosio TG Gosselin RA Gotay CC Goto A Gouda HN Guerrant Rl Gugnani HC Gunnell D Gupta R Gupta R Gutierrez RA Hafezi-Nejad N Hagan H Halasa Y Hamadeh RR Hamavid H Hammami M Hankey GJ Hao Y Harb HL Haro JM Havmoeller R Hay RJ Hay S Hedayati MT Pi IBH Heydarpour P Hijar M Hoek HW Hoffman HJ Hornberger JC Hosgood HD Hossain M Hotez PJ Hoy DG Hsairi M Hu H Hu G Huang JJ Huang C Huiart L Husseini A Iannarone M Iburg KM Innos K Inoue M Jacobsen KH Jassal SK Jeemon P Jensen PN Jha V Jiang G Jiang Y Jonas JB Joseph J Juel K Kan H Karch A Karimkhani C Karthikeyan G Katz R Kaul A Kawakami N Kazi DS Kemp AH Kengne AP Khader YS Khalifa SEA Khan EA Khan G Khang Y-H Khonelidze I Kieling C Kim D Kim S Kimokoti RW Kinfu Y Kinge JM Kissela BM Kivipelto M Knibbs L Knudsen AK Kokubo Y Kosen S Kramer A Kravchenko M Krishnamurthi RV Krishnaswami S Defo BK Bicer BK Kuipers EJ Kulkarni VS Kumar K Kumar GA Kwan GF Lai T Lalloo R Lam H Lan Q Lansingh VC Larson H Larsson A Lawrynowicz AE Leasher JL Lee J-T Leigh J Leung R Levi M Li B Li Y Li Y liang J Lim S Lin H-H Lind M Lindsay MP Lipshultz SE Liu S Lloyd BK Ohno SL Logroscino G Looker KJ Lopez AD Lopez-Olmedo N Lortet-Tieulent J Lotufo PA Low N Lucas RM Lunevicius R Lyons RA Ma J Ma S Mackay MT Majdan M Malekzadeh R Mapoma CC Marcenes W March LM Margono C Marks GB Marzan MB Masci JR Mason-Jones AJ Matzopoulos RG Mayosi BM Mazorodze TT McGill NW McGrath JJ McKee M McLain A McMahon BJ Meaney PA Mehndiratta MM Mejia-Rodriguez F Mekonnen W Melaku YA Meltzer M Memish ZA Mensah G Meretoja A Mhimbira FA Micha R Miller TR Mills EJ Mitchell PB Mock CN Moffitt TE Ibrahim NM Mohammad KA Mokdad AH Mola GL Monasta L Montico M Montine TJ Moore AR Moran AE Morawska L Mori R Moschandreas J Moturi WN Moyer M Mozaffarian D Mueller UO Mukaigawara M Murdoch ME Murray J Murthy KS Naghavi P Nahas Z Naheed A Naidoo KS Naldi L Nand D Nangia V Narayan KMV Nash D Nejjari C Neupane SP Newman LM Newton CR Ng M Ngalesoni FN Nhung NT Nisar MI Nolte S Norheim OF Norman RE Norrving B Nyakarahuka L Oh IH Ohkubo T Omer SB Opio JN Ortiz A Pandian JD Panelo CIA Papachristou C Park E-K Parry CD Caicedo AJP Patten SB Paul VK Pavlin BI Pearce N Pedraza LS Pellegrini CA Pereira DM Perez-Ruiz FP Perico N Pervaiz A Pesudovs K Peterson CB Petzold M Phillips MR Phillips D Phillips B Piel FB Plass D Poenaru D Polanczyk GV Polinder S Pope CA Popova S Poulton RG Pourmalek F Prabhakaran D Prasad NM Qato D Quistberg DA Rafay A Rahimi K Rahimi-Movaghar V Rahman Sur Raju M Rakovac I Rana SM Razavi H Refaat A Rehm J Remuzzi G Resnikoff S Ribeiro AL Riccio PM Richardson L Richardus JH Riederer AM Robinson M Roca A Rodriguez A Rojas-Rueda D Ronfani L Rothenbacher D Roy N Ruhago GM Sabin N Sacco RL Ksoreide K Saha S Sahathevan R Sahraian MA Sampson U Sanabria JR Sanchez-Riera L Santos IS Satpathy M Saunders JE Sawhney M Saylan MI Scarborough P Schoettker B Schneider IJ Schwebel DC Scott JG Seedat S Sepanlou SG Serdar B Servan-Mori EE Shackelford K Shaheen A Shahraz S Levy TS Shangguan S She J Sheikhbahaei S Shepard DS Shi P Shibuya K Shinohara Y Shiri R Shishani K Shiue I Shrime MG Sigfusdottir ID Silberberg DH Simard EP Sindi S Singh JA Singh L Skirbekk V Sliwa K Soljak M Soneji S Soshnikov SS Speyer P Sposato LA Sreeramareddy CT Stoeckl H Stathopoulou VK Steckling N Stein MB Stein DJ Steiner TJ Stewart A Stork E Stovner LJ Stroumpoulis K Sturua L Sunguya BF Swaroop M Sykes BL Tabb KM Takahashi K Tan F Tandon N Tanne D Tanner M Tavakkoli M Taylor HR Te Ao BJ Temesgen AM Have MT Tenkorang EY Terkawi AS Theadom AM Thomas E Thorne-Lyman AL Thrift AG Tleyjeh IM Tonelli M Topouzis F Towbin JA Toyoshima H Traebert J Tran BX Trasande L Trillini M Truelsen T Trujillo U Tsilimbaris M Tuzcu EM Ukwaja KN Undurraga EA Uzun SB van Brakel WH van de Vijver S Dingenen RV van Gool CH Varakin YY Vasankari TJ Vavilala MS Veerman LJ Velasquez-Melendez G Venketasubramanian N Vijayakumar L Villalpando S Violante FS Vlassov VV Waller S Wallin MT Wan X Wang L Wang J Wang Y Warouw TS Weichenthal S Weiderpass E Weintraub RG Werdecker A Wessells KRR Westerman R Wilkinson JD Williams HC Williams TN Woldeyohannes SM Wolfe CD Wong JQ Wong H Woolf AD Wright JL Wurtz B Xu G Yang G Yano Y Yenesew MA Yentur GK Yip P Yonemoto N Yoon S-J Younis M Yu C Kim KY Zaki MES Zhang Y Zhao Z Zhao Y Zhu J Zonies D Zunt JR Salomon JA Murray CJ. Global, regional, and national incidence, prevalence, and years lived with disability for 301 acute and chronic diseases and injuries in 188 countries, 1990–2013: a systematic analysis for the Global Burden of Disease Study 2013. Lancet 2015;386:743–800. 10.1016/S0140-6736(15)60692-4 PMC4561509 26063472

[166] Walker ED Gibbs MT Natoli AR Jones MD. Navigating complexities: clinicians' experiences and systemic challenges in the implementation of evidence-based practice for chronic low back pain – a qualitative study. Disabil Rehabil 2025;47:1697–707. 10.1080/09638288.2024.2378371 39001692

[167] Walker P Morgan SD Day M Blyth F. Commissioning community-based pain programs: A summary of research findings to support Primary Health Networks. Sydney: The University of Sydney and the Australian Prevention Partnership Centre, 2021.

[168] Wallace B Varcoe C Holmes C Moosa-Mitha M Moor G Hudspith M Craig KD. Towards health equity for people experiencing chronic pain and social marginalization. Int J Equity Health 2021;20:53. 10.1186/s12939-021-01394-6 PMC7852178 33531018

[169] Webster F Connoy L Rice K Katz J Sud A Dale C. Chronic struggle: a new framework for understanding chronic pain and marginalization. Social determinants vulnerable populations 2022;20:2965.

[170] Webster F Connoy L Sud A Rice K Katz J Pinto AD Upshur R Dale C. Chronic struggle: an institutional ethnography of chronic pain and marginalization. J Pain 2023;24:437–48. 10.1016/j.jpain.2022.10.004 36252618

[171] Webster F Rice K Katz J Bhattacharyya O Dale C Upshur R. An ethnography of chronic pain management in primary care: the social organization of physicians' work in the midst of the opioid crisis. PLoS One 2019;14:e0215148. 10.1371/journal.pone.0215148 PMC6493733 31042733

[172] Welch V Petticrew M Tugwell P Moher D O'Neill J Waters E White H Group the P-EB. PRISMA-equity 2012 extension: reporting guidelines for systematic reviews with a focus on health equity. PLoS Med 2012;9:e1001333. 10.1371/journal.pmed.1001333 PMC3484052 23222917

[173] Welch VA Norheim OF Jull J Cookson R Sommerfelt H Tugwell P; CONSORT-Equity and Boston Equity Symposium. CONSORT-Equity 2017 extension and elaboration for better reporting of health equity in randomised trials. BMJ 2017;359:j5085. 10.1136/bmj.j5085 29170161

[174] Australian Institute of Health and Welfare. What are determinants of health? 2024. Available at: https://www.aihw.gov.au/reports/australias-health/what-are-determinants-of-health. Accessed June 4, 2025.

[175] What is PRAPARE|PRAPARE. Available at: https://prapare.org/what-is-prapare/. Accessed June 5, 2025.

[176] Wuest J Merritt-Gray M Ford-Gilboe M Lent B Varcoe C Campbell JC. Chronic pain in women survivors of intimate partner violence. J Pain 2008;9:1049–57. 10.1016/j.jpain.2008.06.009 18701353

[177] Yong RJ Mullins PM Bhattacharyya N. Prevalence of chronic pain among adults in the United States. PAIN 2022;163:e328–e332. 10.1097/j.pain.0000000000002291 33990113

[178] Yoshikawa K Brady B Perry MA Devan H. Sociocultural factors influencing physiotherapy management in culturally and linguistically diverse people with persistent pain: a scoping review. Physiotherapy 2020;107:292–305. 10.1016/j.physio.2019.08.002 32026832

[179] Zadro J O'Keeffe M Maher C. Do physical therapists follow evidence-based guidelines when managing musculoskeletal conditions? Systematic review. BMJ Open 2019;9:e032329. 10.1136/bmjopen-2019-032329 PMC6797428 31591090

[180] Zimmer Z Fraser K Grol-Prokopczyk H Zajacova A. A global study of pain prevalence across 52 countries: examining the role of country-level contextual factors. PAIN 2022;163:1740–50. 10.1097/j.pain.0000000000002557 PMC9198107 35027516

